# Keratoconus: Diagnosis and Management by Family Physicians

**DOI:** 10.7759/cureus.86668

**Published:** 2025-06-24

**Authors:** A C Aranda, Sandra S Quitério, Bárbara Junqueira

**Affiliations:** 1 Family Medicine, Unidade de Saúde Familiar (USF) São Domingos, Unidade Local de Saúde (ULS) da Lezíria, Santarém, PRT; 2 Family Medicine, Unidade de Saúde Familiar (USF) Cartaxo Terra Viva, Unidade Local de Saúde (ULS) da Lezíria, Cartaxo, PRT

**Keywords:** corneal diseases, early diagnosis, keratoconus, primary care, visual acuity

## Abstract

Keratoconus is a chronic progressive disease that affects the cornea. Given the nonspecificity of the initial symptoms, it is often confused with common refractive errors (myopia and irregular astigmatism). This is a case of a 29-year-old male patient with a progressive decrease in visual acuity in his right eye, associated with ocular pruritus and frequent eye rubbing. Clinical examination revealed signs suggestive of keratoconus, leading to a timely referral to ophthalmology. Even so, the patient had to undergo a corneal transplant. Despite rapid recognition and referral by a family physician (FP), the impact on quality of life was significant, requiring time off work and support for daily activities. The case illustrates the importance of thorough physical examination and clinical suspicion in Primary Health Care for early diagnosis. It highlights the role of FPs in preventing the progression of the disease and collaborative management with ophthalmology to improve the prognosis and quality of life of patients.

## Introduction

Keratoconus is a chronic, bilateral, asymmetrical, and progressive disease that affects the cornea, leading to a cone-shaped deformation. This deformation can cause visual alterations such as myopia and irregular astigmatism [1,2].

The disease is mainly diagnosed in adolescence or early adulthood, with a prevalence of between 0.2 and 4.79 in 100,000 people and an incidence of between 1.5 and 25 in 100,000 people/year, with no significant differences between the sexes [3].

Although the etiological mechanism is still not fully understood, keratoconus is multifactorial, with a relevant genetic contribution [3,4].

Some of the risk factors studied include personal history of eczema, asthma, and allergies [3], excessive exposure to ultraviolet (UV) radiation, environmental factors such as eye rubbing, repeated trauma, and use of contact lenses, especially rigid lenses [5].

Clinically, the ocular symptoms depend on the severity and progression of the disease [6-9]. At first, the presentation may be discreet (blurred vision, increased sensitivity to light or glare) and may be undervalued by the patient. Common signs that precede ectasia (pathological thinning and protrusion of the cornea, leading to an irregular shape) include an increase in the curvature of the cornea, especially in the inferior and superior regions [9-11].

Some advanced signs of keratoconus, such as Munson's sign (V-shaped deformation of the lower eyelid during downgaze), may be observed without specialized equipment in a primary care setting. However, the gold standard for diagnosing keratoconus remains corneal topography, a non-invasive imaging technique that maps the curvature and shape of the corneal surface. This method produces a detailed, color-coded map that reveals subtle corneal irregularities indicative of the disease. Despite its diagnostic value, corneal topography is typically not available in routine Family Medicine consultations [3,9-14].

The main goals of treatment are to stop the progression of the disease and to achieve visual rehabilitation, and there are no specific guidelines [6,9,10]. Treatment varies according to age, tolerance, severity, and progression: from correction with glasses and contact lenses to interventions such as corneal cross-linking and corneal transplants in severe cases [3]. Most patients do not need a corneal transplant and can adequately control visual acuity deficits with contact lenses [6-9]. Corneal transplantation is offered to 21%-60% of keratoconus patients who are eligible for surgery [9,10].

Progression is faster in younger patients, but keratoconus usually stabilizes by the fourth decade. Recurrence after keratoplasty is rare, occurring in 5-12% of cases, typically 18-21 years later, with signs like astigmatism, scarring, thinning, Vogt's striae (bands visible on slit-lamp biomicroscopy, thought to represent collagen lamellae under mechanical stress), and Munson's sign [3,9,15].

For prevention, high-risk patients should avoid eye rubbing, use preservative-free antihistamines for allergies, and lubricants for irritation, preferably with multiple actions.

Early diagnosis is difficult due to nonspecific symptoms. Keratoconus significantly impacts quality of life, and a family physician (FP) plays a key role in early detection, prevention, education, and referral [6,9,10].

## Case presentation

The patient is a 29-year-old man who is a professional driver with a history of obesity and dyslipidemia, without regular medication and with no relevant family history, including no hereditary ophthalmic diseases or conditions associated with keratoconus, such as eczema, asthma, or allergies.

He consulted his FP in January 2024 for a progressive decrease in visual acuity in his right eye (RE), associated with ocular pruritus and frequent rubbing, which had been going on for around three months. There was no history of ocular trauma.

**Figure 1:**
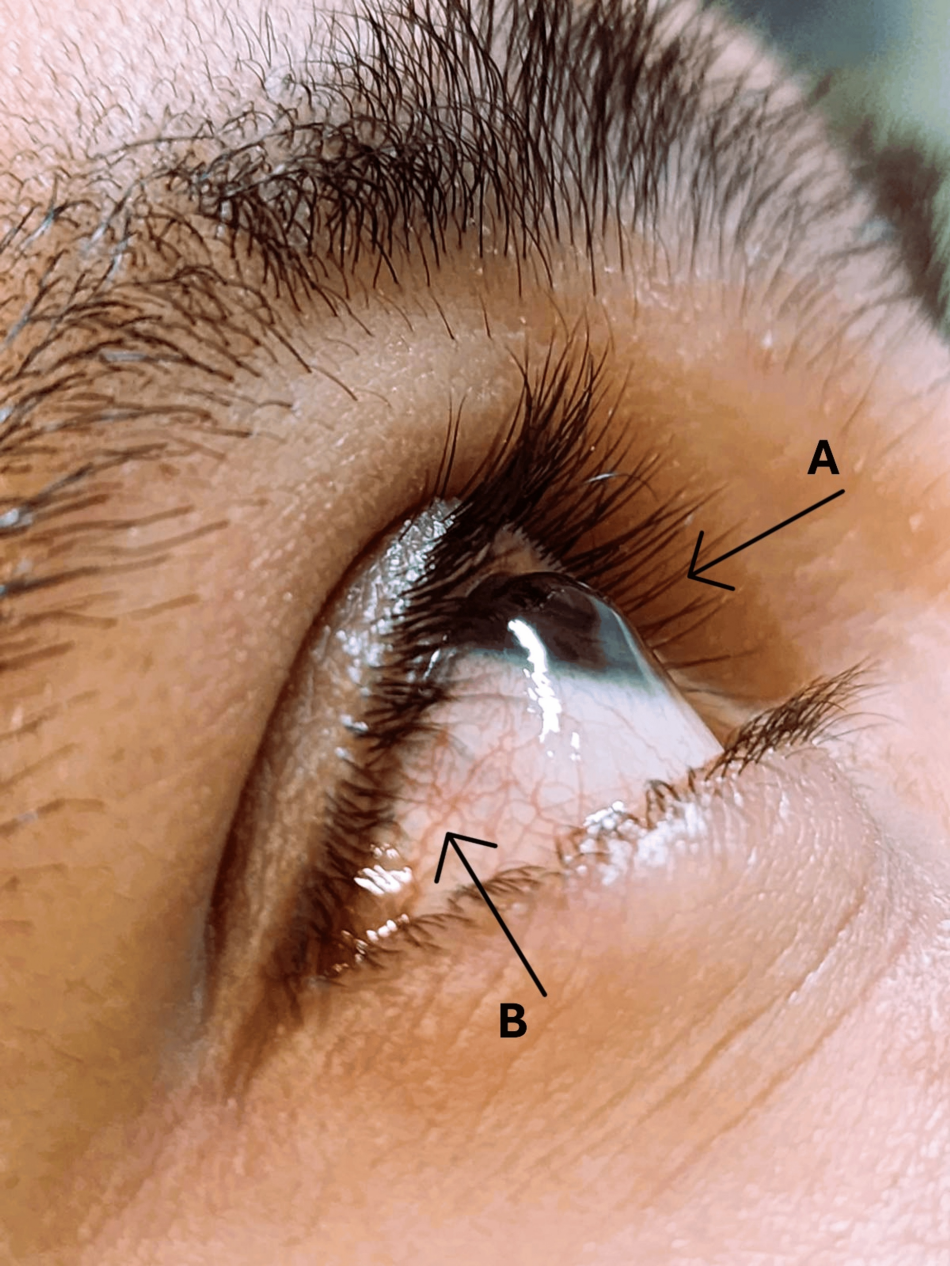
Characteristic keratoconus findings (right eye) A: Corneal protrusion - Munson's sign; B: Conjunctival hyperemia

The initial physical examination showed isochoric and isoreactive pupils, cone-shaped tapering of the cornea, Munson's sign (Figure 1A), conjunctival hyperemia (Figure 1B), and visual acuity of 1/10 in the RE and 9/10 in the left eye (LE).

Allergic complaints were screened using the CARAT questionnaire: 29 points (11/12 in the upper airways and 19/19 in the lower airways), indicating good overall control.

Based on these clinical findings, a diagnosis of keratoconus was made. The patient was referred for an urgent ophthalmology consultation, with the recommendation to avoid eye rubbing. Artificial tears were prescribed for the patient's comfort.

While waiting for his first ophthalmology appointment, the patient went to the emergency department due to worsening complaints of ocular pain in the RE. The ophthalmological assessment revealed uncorrected visual acuity of <0.5/10 in the RE and 2/10 with correction, and in the LE, visual acuity of 5/10 without correction. Biomicroscopy revealed mild conjunctival hyperemia, hydrops, and the presence of keratoconus in the RE (stage 4 - severe), and in the LE, a diagnosis of Keratoconus without hydrops (stage 3 - moderate) was made. Fundoscopy revealed no acute alterations in either eye.

Ophthalmology's diagnostic impression was acute keratoconus hydrops in the RE with low visual acuity. The instituted treatment is summarized in Table 1 [16].

**Table 1:** Pharmacological treatment implemented in the acute management of keratoconus

Medication	Concentration	Dosage	Frequency	Pharmacological Class	Mechanism of Action
Chloramphenicol (ointment)	10 mg/g	1 application	4 times a day	Broad-spectrum antibiotic	Inhibits bacterial protein synthesis by binding to the 50S ribosomal subunit.
Cyclopentolate	10 mg/mL	1 drop	Every 12 hours	Anticholinergic (Mydriatic/Cycloplegic)	Blocks muscarinic receptors in the eye, causing pupil dilation and paralysis of accommodation.
Brimonidine	0.25 mg/mL	1 drop	Every 12 hours	Alpha-2 adrenergic agonist	Reduces aqueous humor production and increases uveoscleral outflow, lowering intraocular pressure.
Artificial tears (Hyaluronic acid)	Not specified	1 drop	Twice a day	Ocular lubricant	Provides moisture and protection to the ocular surface by mimicking natural tears.

The need to avoid eye rubbing was reinforced, and he was referred to a specialist cornea consultation at another hospital as a way of speeding up the process.

The patient’s ophthalmology consultation was ultimately conducted in November of that year and his visual acuity improved following the initial treatment. After bilateral cycloplegia, the RE presented uncorrected visual acuity of 2/10 (stage 4 - severe) and the LE 8/10 (stage 2 - early). A biomicroscopy showed keratoconus in both eyes and acute hydrops in the RE, with no colorable lesion and no infiltrate. He was referred for corneal transplantation of the RE by deep anterior lamellar keratoplasty (DALK), which he underwent in March 2025.

DALK is a surgical technique used to replace diseased recipient stroma with donor corneal stroma, while the recipient corneal endothelium and posterior limiting lamina are retained, preserving the ocular integrity [3].

Two months after surgery, he had no signs of infection or graft rejection, adequate intraocular tension and no alterations on fundoscopy with visual acuity RE 5/10 (stage 3 - moderate) and LE 9/10 (stage 2 - early).

He is currently medicated with dexamethasone 1 mg/ml - one drop 8/8h and cyclosporine 50 mg - 12/12h for two months, per os and artificial tears. A summary of the case progression is presented in Table 2.

**Table 2:** Summary of case progression and clinical findings RE: right eye; LE: left eye; DALK: deep anterior lamellar keratoplasty (surgical procedure performed on the cornea) *Hydrops: A rupture in Descemet’s membrane that allows aqueous humor to enter the corneal stroma. This leads to corneal edema, resulting in a sudden decrease in visual acuity, eye pain, photophobia, and increased light scattering. It may present with corneal opacification seen on slit-lamp examination.

Date	Clinical Event	Visual Acuity (RE / LE)	Clinical Findings
January 2024 - Family Physician consultation	Progressive decrease in RE visual acuity, ocular pruritus, frequent rubbing	RE: 1/10 / LE: 9/10 (both uncorrected)	Physical exam: cone-shaped cornea, Munson’s sign, conjunctival hyperemia. Diagnosed keratoconus.
July 2024 - Emergency department visit	Worsening ocular pain	RE: <0.5/10 (uncorrected), 2/10 (corrected) / LE: 5/10 (uncorrected)	Biomicroscopy: RE hydrops with keratoconus, LE keratoconus; fundoscopy normal; diagnosed acute hydrops* in the RE.
November 2024 - Ophthalmology consultation	Same clinical status	RE: 2/10 (uncorrected, post-cycloplegia) / LE: 8/10	Biomicroscopy confirmed bilateral keratoconus, acute hydrops in the RE.
March 2025 - Corneal transplant	DALK on RE	Same	Uncomplicated surgery.
May 2025	No signs of infection or graft rejection; adequate intraocular pressure; fundoscopy normal	RE: 5/10 / LE: 9/10 (both uncorrected)	Postoperative status stable with improved vision.

Since November, he has also attended multiple specialized corneal ophthalmology appointments to prepare for surgery and, subsequently, to monitor postoperative progress, including screening for rejection, signs of infection, and improvement in visual acuity.

Given the good progress so far and the opinion of the attending ophthalmologist, a complete functional recovery is expected after stabilization of the surgical intervention.

During this period, the patient has been followed monthly by his FP to assess pain levels, monitor visual acuity, and evaluate the impact of the condition on his quality of life. From the first consultation, a certificate of temporary incapacity for work activities was required, as the patient was no longer able to drive, a fundamental condition for his work. He was left with significant day-to-day functional limitations, requiring support in activities that involved reading (from letters and documents to cell phone messages) and handling small objects.

## Discussion

Early detection of keratoconus remains challenging due to the nonspecificity of the initial symptoms, which are often mistaken for simple refractive errors. In the case under study, the patient came to the FP at a time when he already had severe visual acuity impairment (1/10). It is known from the literature that in cases where corrected visual acuity reaches 6/6 without obvious clinical signs of keratoconus, detection is unlikely unless corneal topography is performed [3]. The lack of resources such as corneal topography in Primary Health Care limits the early identification of keratoconus, as shown in this case. Here, the diagnosis was made at an advanced stage, with hydrops in the RE.

The fact that the patient was young may also explain the faster progression of the disease [3]. This diagnosis at an advanced stage meant that, contrary to what is often described in the literature [3], it was necessary to resort to corneal transplantation as a surgical intervention to treat the patient.

This patient had no risk factors such as hereditary eye diseases, genetic syndromes, or connective tissue diseases, contact lens use, eczema, asthma, and allergies, except for the habit of rubbing his eyes. 

Regarding excessive UV radiation exposure, the literature [5,7,9] presents discrepant findings and does not clearly distinguish the roles of UV-A or UV-B in the development of keratoconus. While it is reasonable to consider whether the patient’s occupation might contribute to increased UV exposure, this appears unlikely in his case. His current role as a driver is recent, and he previously worked indoors. As such, it is improbable that he experienced prolonged UV exposure beyond that of the general population, making a work-related etiology for his condition less likely.

This case illustrates the importance of physical examination in routine consultations, especially in patients with visual complaints and eye rubbing habits, even though this patient had no asthma, allergic rhinitis, or other allergies. The role of the FP is crucial in identifying suggestive signs, such as changes in corneal curvature, reduced visual acuity, and irregular reflexes.

With early identification of Keratoconus, immediate referral to Ophthalmology is necessary. However, as exemplified in this case, the difficulty in accessing consultations with this specialty, especially in cornea, highlights the need for support from the FP in the follow-up and monitoring of these patients, in collaborative management.

In this case, it was decided to schedule a monthly appointment (in person or by telephone) to check on the progression of the disease and renew the temporary incapacity certificate (he was absent from work for almost a year and a half).

From a preventive standpoint, the subtle nature of early clinical findings in keratoconus underscores the importance of proactive surveillance, with a focus on identifying risk factors and recognizing epidemiological patterns. FPs play a central role in this effort by promoting patient education on self-care strategies, such as avoiding eye rubbing, and prescribing supportive treatments like antihistamines and artificial tears when appropriate.

FPs can also contribute by conducting opportunistic screenings and fostering close collaboration between community opticians and ophthalmology services, aiming to detect cases before overt signs, such as Munson’s sign, become evident. Facilitating regular follow-up through more frequent in-person visits or teleconsultations (addressing symptoms such as eye pain, sudden visual changes, or daily functional impact) and expediting referral pathways to ophthalmology are crucial steps in preventing disease progression and preserving visual function.

Individuals with a family history of keratoconus are at significantly increased risk of developing the condition themselves, with studies estimating a 15-to-67-fold higher likelihood compared to those without such a history [17]. Given this strong genetic predisposition, it is crucial for FPs to not only manage the affected patient but also to refer first-degree relatives for screening, particularly siblings and children. In this case, the patient’s two brothers are already scheduled for ophthalmologic evaluation, reflecting good preventive practice in Primary Health Care. Early identification through targeted screening may enable timely intervention, potentially preserving visual function and improving long-term outcomes.

## Conclusions

Keratoconus has a significant impact on patients' quality of life, as evidenced in this case by the marked reduction in visual acuity. In addition to compromising basic and instrumental activities of daily living, the pathology affected the patient's work performance, independence, and privacy. This case highlights the role of the FP in the initial recognition and management of a case of keratoconus, helping to minimize the repercussions and improve the prognosis.
